# P-1261. Phamacokinetic Model of Oral Minocycline in Critically Ill Adult Patients with *Acinetobacter baumannii* Infection

**DOI:** 10.1093/ofid/ofae631.1443

**Published:** 2025-01-29

**Authors:** Vasiliki Koumaki, Zoe Athanassa, Georgia Valsami, Paraskevi Papakyriakopoulou, Elmina-Marina Saitani, Silvia Marques, Sofia Manioudaki, Aikaterini Sakagianni, Aristides Dokoumatzidis, Athanasios Tsakris

**Affiliations:** National and Kapodistrian University of Ahens, Athens, Attiki, Greece; Sismanoglio Hospital, Athens, Greece, Athens, Attiki, Greece; National and Kapodistrian University of Ahens, Athens, Attiki, Greece; National and Kapodistrian University of Ahens, Athens, Attiki, Greece; National and Kapodistrian University of Ahens, Athens, Attiki, Greece; Universitas Miguel Hermandez, Valencia, Comunidad Valenciana, Spain; Sismanogleio General Hospital, Athens, Attiki, Greece; Sismanogleio General Hospital, Athens, Attiki, Greece; National and Kapodistrian University of Ahens, Athens, Attiki, Greece; National and Kapodistrian University of Athens, Athens, Attiki, Greece

## Abstract

**Background:**

Minocycline has made a comeback in the drug armamentarium as a treatment option for infections due to *Acinetobacter baumannii*. Although, it has been launched in the 1960s, scarce pharmacokinetic studies have been published. This open-label, prospective clinical study was conducted in critical ill patients with documented infections, mainly VAP-associated pneumonia, attributed to extensively-drug or pan-drug resistant *Acinetobacter baumannii*, susceptible-or intermediate susceptible to minocycline strains, treated with oral minocycline as a combination therapy.

PTA of 200mg and 400mg of minocycline for a range of MICs after a Monte Carlo simulation of 10000 AUC values

**Figure:**
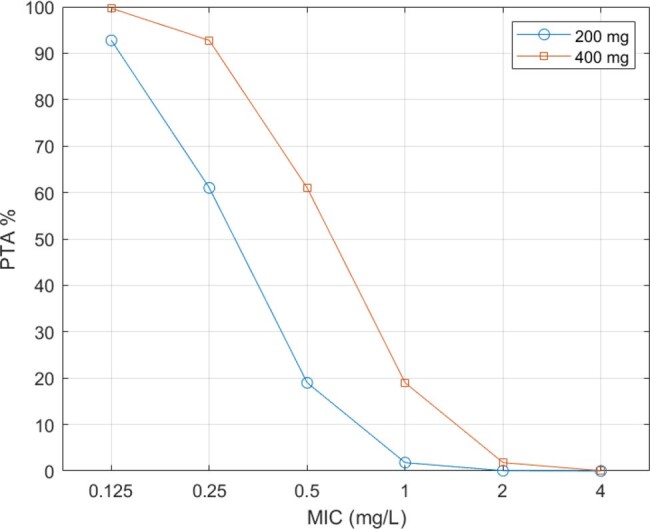

**Methods:**

The PK study population consisted of 20 patients, hospitalized in the ICU of a tetriary Hospital in Athens, Greece. The minimum inhibitory concentration was determined using broth microdilution and a susceptibility breakpoint of ≤ 4mg/L was applied for interpretation, according to the Clinical and Laboratory Standards Institute (CLSI). All patients were given a loading dose of 200mg of minocycline followed by 100mg every 12 hours. Plasma PK samples were collected predose and at 13,14,18,21.5, 59.5,61, 64,68 and 71.5 hours after commencement of minocycline. Minocycline quantification in patient serum sample was conducted using HPLC-PDA, Shimadzu Prominence system (Shimadzu, Kyoto, Japan). A population pharmacokinetic model was developed in Monolix 2023 R1. Monte Carlo simulations were carried out, assuming protein binding of 76%. The probability of target attainment was calculated for each MIC value to achieve an fAUC/MIC ratio of above 25 for daily doses of 200mg and 400mg.

**Results:**

MInocycline PK parameters in critical ill adult patients were best described using a one-compartment model. The PTA values for various MICs are shown in Figure 1, which indicates that an oral dosage of 200mg and 400mg daily, could cover infections, due to *Acinetobacter baumannii* strains exhibiting MICs of 0.125mg/L and 0.25 mg/L ,respectively, with a PTA >90%.

**Conclusion:**

These findings suggest that current oral treatment may be suboptimal for treating such infections, at least as a monotherapy. Further research is needed in order to explore possible dose elevations and synergistic combinations that could enhance its efficacy in combatting XDR, and PDR *Acinetobacter baumannii* infections.

**Disclosures:**

**All Authors**: No reported disclosures

